# Elevated serum IFN-γand IFN-γ/IL-6 ratio in Kikuchi-Fujimoto disease

**DOI:** 10.1186/s12969-023-00877-w

**Published:** 2023-08-22

**Authors:** Tingyan He, Zixuan Shen, Jiayun Ling, Xiaona Zhu, Jun Yang

**Affiliations:** Department of Rheumatology and Immunology, Shenzhen Children’s Hospital, 7019 Yitian Road, Shenzhen, 518038 China

**Keywords:** Kikuchi-Fujimoto disease, Histiocytic necrotizing lymphadenitis, Systemic juvenile idiopathic arthritis, Kawasaki disease, Macrophage activation syndrome

## Abstract

**Background:**

Kikuchi-Fujimoto disease (KFD) is typically a benign, self-limiting inflammatory disease. The diagnosis of KFD can be challenging for nonspecific symptoms, laboratory or imaging findings. In this study, we aimed to describe the clinical manifestations of patients with KFD and to access the potential role of serum cytokines in the diagnosis of this disease.

**Methods:**

Patients with KFD were retrospectively enrolled from January 2015 to November 2021 at Shenzhen Children’s Hospital. Clinical data were collected from inpatient or outpatient medical records. Serum cytokines were detected by the Flowcytomix technique. Serum levels of cytokines were compared between patients with KFD and SJIA, or patients with KFD and KD. The data of patients without MAS were further analyzed. A receiver operating characteristic (ROC) curve analysis was further performed to access the potential role of serum cytokines in the diagnosis of KFD.

**Results:**

Serum cytokines were detected in 25 (43.8%, 25/57) patients with a histological diagnosis of KFD. Compared to SJIA or KD patients, the KFD group had a significantly higher IFN-γ/IL-6 ratio and much lower levels of serum IL-6. The median level of serum IFN-γ in KFD was 41.65 pg/ml (range, 21.04–70.74 pg/ml), which was much higher than that in SJIA (median: 3.33 pg/ml, *p* = 0.16) or KD (median: 2.6 pg/ml, *p* = 0.01). After excluding patients with MAS, there was statistical significance in all comparisons of serum IFN-γ, IFN-γ/IL-6 ratio, and serum IL-6. The cutoff values of serum IFN-γ, IL-6, and IFN-γ/IL-6 ratio for differentiating KFD from SJIA were > 8.48 pg/ml, < 47.42 pg/ml, and > 0.45, respectively. The cutoff values of serum IFN-γ, IL-6, and IFN-γ/IL-6 ratio for differentiating KFD from KD were > 8.56 pg/ml, < 50.45 pg/ml, and > 0.45, respectively. The specificity of all those cutoff values for differentiating KFD from SJIA or KD was ≥ 94.7%.

**Conclusions:**

For patients with fever of unknown etiology and lymphadenopathy, after excluding HLH or MAS, serum IFN-γ > 8.56 pg/mL and IFN-γ/IL-6 ratio > 0.45 may highly suggest the diagnosis of KFD; serum IL-6 > 50.45 pg/mL indicates that the probability of KFD may be small, and sJIA, KD, and acute infection should be excluded first.

**Supplementary Information:**

The online version contains supplementary material available at 10.1186/s12969-023-00877-w.

## Introduction

Kikuchi-Fujimoto disease (KFD) is a benign, self-limiting inflammatory disease characterized by fever and cervical lymphadenopathy. The diagnosis of KFD can be challenging since symptoms, laboratory and imaging findings in this disease are non-specific. KFD can frequently be mistaken for other diseases [1, 2]. Differential diagnoses include infectious lymphadenitis, malignancies such as lymphoma, and autoimmune/autoinflammatory diseases such as systemic lupus erythematosus (SLE), Systemic juvenile idiopathic arthritis (SJIA), and Kawasaki disease.

Although KFD is typically self-limited, some patients may have a prolonged or recurrent disease course, or even present with life-threatening complications such as hemophagocytic Lymphohistiocytosis(HLH) [3, 4]. Thus the accurate and timely diagnosis of this disease will be quite important in proper treatments and improving the prognosis of patients. Although histopathological diagnosis by a lymph node biopsy is crucial to differentiate KFD from other etiologies, it is invasive and may share overlapping histopathological features with other diseases [5, 6]. Therefore, biomarkers by rapid and non-invasive detection will be required in the early diagnosis of KFD.

IFN-γ are rarely encountered in non-specific lymphadenitis tissues, but are frequently detected in the surrounding dead tissue in KFD [7]. Serum levels of IFN-γ and IL-6 are reported to be elevated during the acute phase in KFD [8]. However, the potential role of serum cytokines in the diagnosis and differential diagnosis of this disease remains unknown. Differences from other inflammatory diseases are required to be explored.

Here, we performed a single, retrospective study to describe the clinical manifestations of patients with KFD and to access the potential role of serum cytokines in the diagnosis of this disease.

## Patients and methods

### Study population and design

Patients with KFD were retrospectively enrolled from January 2015 to November 2021 at Shenzhen Children’s Hospital. The study was approved by the ethics committee of the hospital. Written informed consent was obtained from all patients’ legal guardians. All patients with KFD were confirmed based on a typical histological diagnosis. Exclusion criteria included patients with a possible alternative diagnosis such as autoimmune diseases (SLE, Sjogren's disease, etc.), autoinflammation diseases (Kawasaki disease, SJIA, etc.), leukemia, lymphoma, lymphoproliferative diseases, or active infection.

The age-matched control groups included all the patients with SJIA or Kawasaki disease (KD) who met the inclusion criteria within the same study period. KD patients less than five years old were excluded to minimize the influence of age factors. Serum cytokines in three groups were all detected before treatment and after fever (≥ 38.5℃) caused by disease activity for at least 72 h by the Flowcytomix technique in the control groups. SJIA was diagnosed following the 2001 International League Against Rheumatism criteria [9, 10]. KD was diagnosed based on the diagnosis criteria provided by the American Heart Association in 2017 [11]. Macrophage activation syndrome (MAS) was diagnosed according to the 2016 EULAR/ACR/PRINTO classification criteria [12]. There were 31 SJIA patients and 27 KD patients fulfilling the inclusion criteria as the control groups, including six with SJIA-MAS.

### Data collection

Clinical data were collected from inpatient or outpatient medical records. Data collected included clinical manifestations, laboratory findings, and levels of serum cytokines. Clinical symptoms and laboratory data were collected at the onset of KFD. Clinical manifestations were listed in Table 1. Laboratory variables included total leukocyte, neutrophils, lymphocyte and platelet counts, erythrocyte sedimentation rate (ESR), levels of hemoglobulin, C-reactive protein (CRP), aminotransferases, albumin, ferritin, fibrinogen, lactate dehydrogenase (LDH), and auto-antibodies such as anti-nuclear antibodies (ANA). Serum cytokines included IFN-γ, IL-10, IL-6, TNF-a, IL-4, and IL-2.

**Table 1:** Clinical and labortatory features of patients with KFD

Characteristics	KFD (*n* = 57)	References
**Demorgraphic Data**		
Age, years	9.08 (6.92–12.17)	
Male to female ratio	2:1 (38: 19)	
**Clinical manifestations**		
Fever	53 (92.98)	
Lymphadenopathy	54 (95.7)	
Hepatomegaly	20 (35.09)	
Splenomegaly	9 (15.79)	
Skin rash	15 (26.31)	
Arthralgia/arthritis	1 (1.75)	
Gastrointestinal symptoms	5 (8.77)	
Respiratory symptoms	7 (12.28)	
Central nervous symptoms	9 (15.79)	
**Laboratory variables**		
WBC count (/μL)	3530 (2540–4620)	4300–11300
Hemoglobin (g/dL)	11.5 (10.6–12.8)	118–156
Neutrophil count (/μL)	1575 (1230–2500)	1600–7800
Lymophocyte count (/μL)	1415 (1060–1820)	1500–4600
Platelet count (× 10^3^/μL)	203 (174–259)	167–453
AST (IU/L)	37 (28–53)	14–44
ALT (IU/L)	21 (12–39)	7–30
Albumin (g/L)	38.6 (35.6–40.4)	39–54
Ferritin (ng/mL)	312.5 (147–710.8)	14–200
Triglyceride (mg/dl)	1.15 (0.96–1.5)	0–1.7
Fibrinogen (mg/dL)	3.78 (3.09–4.27)	1.3–4.5
CRP (mg/L)	5 (1.3–11.9)	0–10
ESR (mm/h)	40 (20–59)	0–15
LDH (IU/mL)	431 (335–647)	192–321
Positive autobodies	10 (17.5)	

Data expressed as median (interquartile range) or n (%)

*KFD* Kikuchi-Fujimoto disease, *WBC* white blood cell, *ESR* erythrocyte sedimentation rate, *CRP* C-reactive protein, *ALT* alanine aminotransferase, *LDH* lactate dehydrogenase, *AST* aspartate aminotransferase, ANA anti-nuclear antibodies

### Statistical analysis

Continuous variables were presented as medians with interquartile ranges (IQR), and categorical variables were presented as frequencies and percentages. Unpaired Welch’s t-test for continuous data and Man-Whitney U test for categorical variables were performed. Differences in clinical features and laboratory findings were analyzed between patients with serum cytokines data and others. Serum levels of cytokines were compared between patients with KFD and SJIA, or patients with KFD and KD. The data of patients without MAS were further analyzed. We subsequently performed a receiver operating characteristic (ROC) curve analysis for the data with statistical differences between the two groups. Analysis was completed with GraphPad Prism 8.0 statistical software (GraphPad Software Inc., La Jolla, CA, USA). A *p*-value of < 0.05 was considered statistically significant.

## Results

### The clinical characteristics of KFD patients

We identified 57 patients with a histological diagnosis of KFD in Shenzhen Children’s Hospital, who were from the same cohort reported previously [13]. Of those, 15 patients had MAS. 25 diagnosed KFD since 2019 had serum cytokines measured, including six with KFD-MAS. The cytokine levels were not measured in the other patients. Clinical characteristics were summarized and compared in Table 1. The median age at diagnosis was 9.08 years (range, 6.92–12.17 yr). Male to female ratio in this study was 2:1. General clinical characteristics included lymphadenopathy (*n* = 54), fever (*n* = 53), hepatomegaly (*n* = 20), skin rash (*n* = 15), splenomegaly (*n* = 9), central nervous (*n* = 9) and respiratory symptoms (*n* = 7). Gastrointestinal symptoms and arthralgia/arthritis were present in five and one patient, respectively. There was no statistical difference in the clinical characteristics of KFD between patients with serum cytokine detection and patients without serum cytokine detection (Supplementary Table 1).

Abnormal laboratory findings included elevated erythrocyte sedimentation rate (median:40 mm/h, range: 20–59 mm/h), mild cytopenia (median:3530/μL, range: 2540–4620/μL), mild lymphopenia (median: 1415/μL, range: 1060–1820/μL), elevated levels of LDH (median: 431 IU/mL, range: 335-647 IU/mL) and ferritin (median: 312.5 ng/mL, range: 147–710.8 ng/mL). Ten patients had presented with positive auto-antibodies, including antinuclear antibody (ANA, *n* = 5), anti-SSA antibody (*n* = 2), anti-RNP antibody (*n* = 3), and anti-DNA antibody (*n* = 1).

### Comparison of serum cytokine levels in patients with KFD, SJIA, or KD

**Fig. 1:**
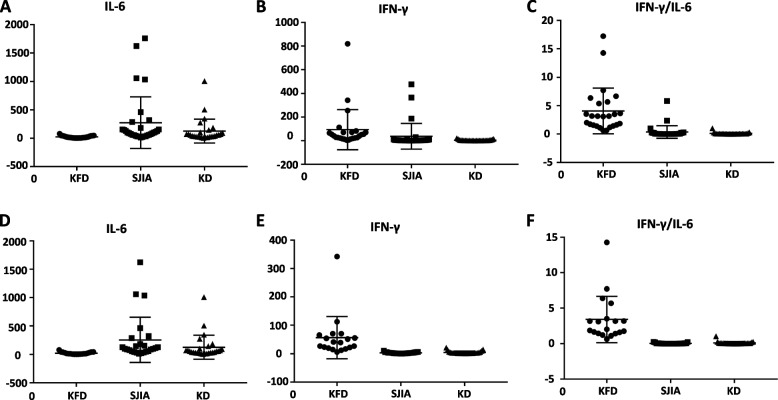
Serum cytokines levels of patients in KFD, SJIA, and KD. Serum IL-6, serum IFN-γ, and IFN-γ/IL-6 ratio in all patients (**A**, **B**, and **C**) and those without MAS (**D**, **E**, and **F**). Differences were analyzed between patients with KFD and SJIA, KFD and KD. Compared to patients with SJIA or KD, patients with KFD showed an elevation of serum IFN-γ and IFN-γ/IL-6 ratio, and a much lower level of serum IL-6. *P*-value was 0.16 for the comparison of serum IFN-γ between all KFD and SJIA patients (**B**). *P*-values for other comparisons were < 0.05

Compared to SJIA patients in active disease, the KFD group had a significantly higher IFN-γ/IL-6 ratio (*p* = 0.0001), and much lower levels of serum IL-6 (*p* = 0.004) and IL-2 (*p* = 0.008). Although there was no statistical difference, the median serum IFN-γ level in KFD was 41.65 pg/ml (range: 21.04–70.74 pg/ml), which was much higher than that in SJIA (Table 2 and Fig. 1A-C). There were no significant differences in other serum cytokines, including IL-10, TNF-a, and IL-4 (Table 2).

**Table 2:** Serum cytokine levels in KFD, SJIA, and KD

	KFD (*n* = 25)	SJIA (*n* = 31)	KD (*n* = 27)	*p-value	**p-value
IFN-γ (pg/ml)	41.65 (21.04–70.74)	3.33 (1.3–6.27)	2.6 (1.46–4.82)	0.16	**0.01**
IL-10 (pg/ml)	5.5 (4.04–8.97)	4.79 (3.81–10.9)	8.54 (5.45–12.17)	0.57	0.22
IL-6 (pg/ml)	17.17 (6.65–40.62)	99.66 (49.19–181.8)	55.26 (26.02–103.52)	**0.004**	**0.019**
IFN-γ/IL-6	3.16 (1.54–5.5)	0.03 (0.01–0.19)	0.05 (0.03–0.08)	**0.0001**	**< 0.0001**
TNF-a (pg/ml)	0.9 (0.28–2.14)	0.82 (0.3–1.82)	2.23 (1.3–2.93)	0.93	0.14
IL-4 (pg/ml)	2.2 (1.43–2.87)	2.04 (1.22–3.16)	2.85 (2.18–3.72)	0.34	**0.01**
IL-2 (pg/ml)	2.17 (0.9–3.95)	3.23 (0.6–9.99)	2.25 (1.21–3.89)	**0.008**	0.13

Data expressed as median (interquartile range). Variables were analyzed by Unpaired Welch’s t-test

*KFD* Kikuchi-Fujimoto disease, *SJIA* Systemic Juvenile Idiopathic Arthritis, *KD* kawasaki disease, *MAS* macrophage activation syndrome

^*^p-value represented difference between patients with KFD and SJIA

^**^p-value was comparison between patients with KFD and KD

Compared to KD patients, the KFD group had a significantly higher serum IFN-γlevel (*p* = 0.01) and IFN-γ/IL-6 ratio (*p* < 0.0001), and much lower levels of serum IL-6 (*p* = 0.019) and IL-4 (*p* = 0.01) (Table 2 and Fig. 1A-C). There were no significant differences in other serum cytokines, including IL-10, TNF-a, and IL-2 (Table 2).

### Comparison of serum cytokine levels in patients without MAS

To exclude the influence of MAS, serum cytokine levels in patients without MAS were further analyzed. Compared to SJIA patients without MAS, the KFD group had a significantly higher serum IFN-γ level (*p* = 0.006) and IFN-γ/IL-6 ratio (*p* = 0.0003), and much lower levels of serum IL-6 (*p* = 0.007) and IL-2 (*p* = 0.016). There were no significant differences in other serum cytokines, including IL-10, TNF-a, and IL-4 (Table 3 and Fig. 1D-F). For the patients with MAS, there were no statistical differences in levels of those serum cytokines between the two groups (Table 3).

**Table 3:** Serum cytokine levels of patients in KFD, SJIA, and KD

Without MAS						With MAS		
	KFD (*n* = 19)	SJIA (*n* = 25)	KD (*n* = 27)	*p-value	**p-value	KFD (*n* = 6)	SJIA (*n* = 6)	***p-value
IFN-γ (pg/ml)	39.3 (19.6–65.4)	2.8 (1.1–4.4)	2.6 (1.46–4.82)	**0.006**	**0.007**	76.9 (29.5–255.8)	108.3 (15.4–365.8)	0.85
IL-10 (pg/ml)	4.4 (3.9–7.7)	4.2 (3.2–6.0)	8.54 (5.45–12.17)	0.72	0.08	15.2 (5.5–22.8)	19.3 (10.9–33.9)	0.36
IL-6 (pg/ml)	13.4 (6.2–37.9)	102 (52.1–234.2)	55.26 (26.02–103.52)	**0.007**	**0.018**	21.3 (9.1–47.6)	65.1 (32.0–154.4)	0.3
IFN-γ/IL-6	2.0 (1.4–3.5)	0.02 (0.008–0.05)	0.05 (0.03–0.08)	**3E-04**	**0.0003**	4.5 (3.5–6.7)	0.63 (0.21–2.37)	0.12
TNF-a (pg/ml)	0.87 (0.12–1.83)	0.82 (0.33–1.78)	2.23 (1.3–2.93)	0.41	**0.032**	2.02 (0.46–3.21)	0.83 (0.19–2.34)	0.33
IL-4 (pg/ml)	2.2 (1.43–3.22)	2.06 (1.08–3.10)	2.85 (2.18–3.72)	0.61	**0.028**	2.07 (1.73–2.36)	1.82 (1.3–4.05)	0.42
IL-2 (pg/ml)	1.4 (0.6–3.19)	3.23 (0.6–9.99)	2.25 (1.21–3.89)	**0.016**	0.07	2.86 (2.14–4.63)	5.44 (0.1–14.62)	0.27

Data expressed as median (interquartile range). Variables were analyzed by Unpaired Welch’s t-test

*KFD* Kikuchi-Fujimoto disease, *SJIA* Systemic Juvenile Idiopathic Arthritis, *KD* kawasaki disease, *MAS* macrophage activation syndrome

^*^p-value and **p-value represented differences between patients with KFD and SJIA, KFD and KD, respectively

^***^p-value was comparison between patients with KFD and SJIA with MAS

Compared to KD patients, the KFD group without MAS had a significantly higher serum IFN-γ level (*p* = 0.007) and IFN-γ/IL-6 ratio (*p* = 0.0003), and much lower levels of serum IL-6 (*p* = 0.018), TNF-a (*p* = 0.032), and IL-4 (*p* = 0.028) (Table 3 and Fig. 1D-F). There were no significant differences in serum levels of IL-10 and IL-2 (Table 3).

### The potential role of serum cytokines in the diagnosis of KFD

**Fig. 2:**
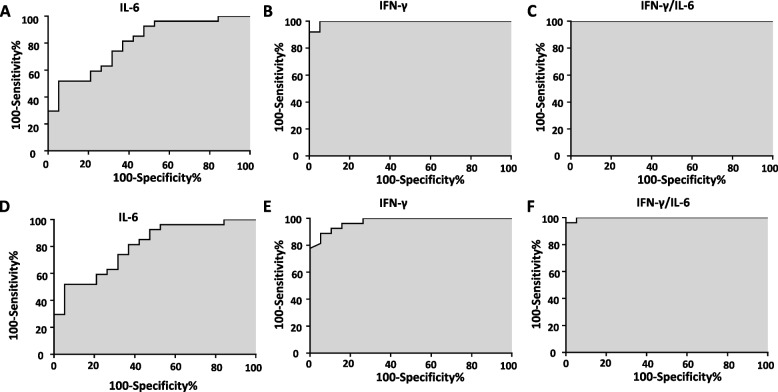
Receiver operating characteristic curves of serum cytokines. The role of serum IL-6, serum IFN-γ, and IFN-γ/IL-6 ratio in the differential diagnosis of KFD from SJIA without MAS (**A**, **B**, and **C**) and KD (**D**, **E**, and **F**)

A ROC curve analysis was performed to access the potential role of serum cytokines in the diagnosis of KFD. The cutoff values of serum IFN-γ, IL-6, IFN-γ/IL-6 ratio, and IL-2 for differentiating KFD from SJIA were > 8.48 pg/ml (AUC:0.99, 95%CI: 0.98–1.0, *p* < 0.0001), < 47.72 pg/ml(AUC:0.92, 95%CI: 0.84–0.99, *p* < 0.0001), > 0.45 (AUC:1, 95%CI: 1.0–1.0, *p* < 0.0001), and < 5.25 pg/ml pg/ml (AUC:0.64, 95%CI: 0.48–0.80, *p* = 0.1), respectively. Except for serum IL-2, both sensitivity and specificity of those cutoff values for identifying these two diseases were ≥ 80% (Table 4 and Fig. 2).

**Table 4:** Receiver operating characteristic curve analysis of serum cytokines in KFD, SJIA, and KD

**Compared to SJIA patients without MAS**						
	AUC	Cut-off Value	Sensitivity (%)	Specificity (%)	95%CI	*p*-value
IFN-γ (pg/ml)	0.99	> 8.48	96	94.7	0.98–1.0	< 0.0001
IL-6 (pg/ml)	0.92	< 47.72	80	94.7	0.84–0.99	< 0.0001
IFN-γ/IL-6	1	> 0.45	100	100	1.0–1.0	< 0.0001
IL-2 (pg/ml)	0.64	< 5.25	36	94.7	0.48–0.80	0.1
**Compared to patients with KD**						
	AUC	Cut-off Value	Sensitivity (%)	Specificity (%)	95%CI	*p*-value
IFN-γ (pg/ml)	0.97	> 8.56	85.2	94.74	0.94–1.0	< 0.0001
IL-6 (pg/ml)	0.8	< 50.45	51.9	94.7	0.67–0.93	0.0006
IFN-γ/IL-6	0.998	> 0.45	96.3	100	0.99–1.0	< 0.0001
IL-2 (pg/ml)	0.63	< 5.25	22.2	94.7	0.47–0.79	0.13

Patients with MAS were all excluded for ROC analysis

*AUC* area under the receiver operating characteristic curve, KFD Kikuchi-Fujimoto disease, SJIA Systemic Juvenile Idiopathic Arthritis, *KD* kawasaki disease, *MAS* macrophage activation syndrome

The cutoff values of serum IFN-γ, IL-6, IFN-γ/IL-6 ratio, and IL-2 were > 8.56 pg/ml (AUC:0.97, 95%CI: 0.94–1.0, *p* < 0.0001), < 50.45 pg/ml (AUC:0.8, 95%CI:0.67–0.93, *p* = 0.0006), > 0.45 (AUC:0.998, 95%CI: 0.99–1.0, *p* < 0.0001), and < 5.25 pg/ml (AUC:0.63, 95%CI: 0.47–0.79, *p* = 0.13), respectively. Except for the sensitivity of serum IL-6 and IL-2, both sensitivity and specificity of other cutoff values for differentiating KFD from KD were ≥ 85.2% (Table 4 and Fig. 2).

## Discussion

As reported previously, clinical features and laboratory findings in this cohort were non-specific [14]. The most common manifestations were fever, cervical lymph node enlargement, skin rash, hepatosplenomegaly, headache, dizziness, mild cough, abdominal pain, and vomiting. Arthralgia/arthritis and oral ulcer were occasionally seen. Abnormal laboratory findings with variable occurrence could be found in KFD, including elevated erythrocyte sedimentation rate, mild cytopenia, mild lymphopenia, hyperferritinemia, elevated levels of LDH, and positive auto-antibodies.

IL-6 is synthesized by myeloid cells, such as macrophages and dendritic cells. Rapid production of IL-6 contributes to host defense during infection and tissue injury, but excessive IL-6 synthesis is involved in the pathogenesis of various diseases, such as SJIA, KD, Castleman disease, COVID-19 infection, etc. [15]. Four patients with KFD had increased serum IL-6 level during the acute phase [8]. More than half of the patients with KFD in this study also showed an increased level of serum IL-6 (60%, 15/25). Although serum IL-6 level in KFD was elevated, the increase was not as obvious as that in SJIA or KD. Serum IL-6 level in most patients with KFD (96%, 24/25) was less than 47.72 pg/ml, which was lower than that in SJIA or KD group. Thus the extremely higher level of serum IL-6 (> 47.72 pg/ml) may largely help to exclude the diagnosis of KFD. The role of IL-6 in the pathogenesis of KFD remains unclear. Further studies are required to explore its association with the pathogenesis of KFD.

Most patients with KFD in our cohort showed an increased serum IFN-γ level (96%, 24/25) and IFN-γ/IL-6 ratio. Serum IFN-γ level in most KFD patients (96%, 24/25) was > 8.56 pg/ml. And the IFN-γ/IL-6 ratio in all of them was > 0.45. Both were extremely higher than that in the other groups without MAS. However, for patients with MAS, our data showed no statistical differences in levels of serum cytokines or in IFN-γ/IL-6 ratio between patients KFD and SJIA. Therefore, for patients without MAS, serum IFN-γ level > 8.56 pg/ml combined with the IFN-γ/IL-6 ratio > 0.45 may help to exclude SJIA or KD, supporting the diagnosis of KFD.

IFN-γ predominantly produced by NK and T cells is a pleiotropic cytokine with multiple effects on the inflammatory response and on innate and adaptive immunity. Aberrant production of IFN-γ underlies a number of hyperinflammatory or immune-mediated diseases, such as primary hemophagocytic lymphohistiocytosis (HLH), various forms of secondary HLH, including MAS, and cytokine release syndrome [16, 17].  The statistical differences especially in serum IFN-γ level were lost in MAS-KFD versus SJIA-MAS. Compared to other serum cytokine levels, serum IFN-γ level is extremely over-secreted in patients with SJIA-MAS, which might eliminate some differences especially in serum IFN-γ level and IFN-γ/IL-6 ratio between two groups. Although IFN-γ was over-produced in most KFD patients, secondary HLH was relatively rare in this disease. Thus the role of IFN-γ in the pathogenesis of KFD and HLH/MAS might be quite different. Further studies including enough patients is required to clarify this phenomenon and reveal the underlying mechanism.

The limitations of this study are the small sample size in a single center and the that it is retrospective. Serum cytokines were not detected in more than half of KFD patients. Except for SJIA and KD, serum cytokines were not checked in most patients with other differential diseases, such as infectious lymphadenitis, lymphoma, etc. Future prospective studies with large sample sizes and more control groups may further explore the exact role of serum IFN-γ, IL-6, and IFN-γ/IL-6 ratio in the KFD diagnosis.

## Conclusions

For patients with fever of unknown etiology and lymphadenopathy, after excluding HLH or MAS, serum IFN-γ > 8.56 pg/mL and IFN-γ/IL-6 ratio > 0.45 may highly suggest the diagnosis of KFD. Serum IL-6 > 50.45 pg/mL indicates that the probability of KFD may be small, and sJIA, KD, and infection should be excluded first.

### Supplementary Information

**Additional file 1: Supplementary Table 1.** Clinical features of KFD patients with or without cytokine detection.

## Data Availability

The datasets generated and/or analyzed during the current study are available from the corresponding author on reasonable request.

## Abbreviations

KFD: Kikuchi-Fujimoto disease
SLE: Systemic lupus erythematosus
SJIA: Systemic juvenile idiopathic arthritis
KD: Kawasaki disease
MAS: Macrophage activation syndrome
AST: Aspartate aminotransferase
ESR: Erythrocyte sedimentation rate
CRP: C-reactive protein
LDH: Lactate dehydrogenase
IVIG: Intravenous immunoglobulin
IQR: Interquartile ranges
ROC: Receiver operating characteristic curve
